# Whole-Genome Sequence of *Pantoea americana* Strain VS1, an Extended-Spectrum β-Lactamase-Producing Epibiont Isolated from *Magnolia grandiflora*

**DOI:** 10.1128/genomeA.01285-17

**Published:** 2017-11-16

**Authors:** Pushpa Lata, Subramaniam S. Govindarajan, Feng Qi, Jian-Liang Li, Santosh K. Maurya, Malaya K. Sahoo

**Affiliations:** a Department of Microbiology and Molecular Biology, University of Central Florida, Orlando, Florida, USA; b Analytical Genomics Core, Sanford Burnham Prebys Medical Discovery Institute at Lake Nona, Orlando, Florida, USA; c Applied Bioinformatics Core, Sanford Burnham Prebys Medical Discovery Institute at Lake Nona, Orlando, Florida, USA; d Sanford Burnham Prebys Medical Discovery Institute at Lake Nona, Orlando, Florida, USA; e Department of Pathology, Stanford University School of Medicine, Palo Alto, California, USA

## Abstract

*Pantoea americana* strain VS1, an extended-spectrum β-lactamase-producing epibiont, was isolated from *Magnolia grandiflora* in central Florida, USA. Here, we report the *de novo* whole-genome sequence of this strain, which consists of a total of 191 contigs spanning 5,412,831 bp, with a GC content of 57.3% and comprising 4,836 predicted coding sequences.

## GENOME ANNOUNCEMENT

Earlier, we published the whole-genome sequence of *Pantoea latae* strain AS1, which was isolated from the rhizosphere of a cycad, *Zamia floridana* (1). Many members of the genus *Pantoea* have been used as a biocontrol agent against some plant pathogens and also for bioremediation and degradation of toxic herbicides (2, 3). In this study, novel *Pantoea americana* strain VS1, an extended-spectrum β-lactamase-producing epibiont, was isolated from *Magnolia grandiflora* in central Florida, USA. Here, we report the *de novo*-sequenced whole genome of *Pantoea americana* strain VS1. The genomic DNA was isolated using the QIAamp DNA minikit (Qiagen, Germantown, MD), according to the manufacturer’s instructions. DNA quality was checked by NanoDrop spectrophotometer (Thermo Scientific) and quantitated by a Qubit 2.0 fluorometer (Life Technologies) and Agilent 2100 Bioanalyzer (Agilent Technologies, Santa Clara, CA). The genomic DNA was fragmented and tagged with sequencing adapters in a single-tube enzymatic reaction using a Nextera XT DNA library preparation kit (Illumina, San Diego, CA). The resulting library was sequenced using the Illumina MiSeq sequencing platform (Illumina, San Diego, CA) and generated 2.9 million reads for *Pantoea americana* strain VS1. The reads were assembled using Minia version 2.0.3 with default parameters and a k-mer size of 121 (4). A total of 191 contigs were obtained, and the *N*_50_ value of the contigs was 151,591 bp. The maximum length of the contig was 601,327 bp, with a total genome length of 5,412,831 bp. The GC content of the genome was 57.3%. The protein-coding regions were predicted by the NCBI Prokaryotic Genome Annotation Pipeline (release 2013; https://www.ncbi.nlm.nih.gov/genome/annotation_prok/). In total, 4,836 coding genes were identified in the 5.4-Mb genome of *Pantoea americana*, and it shows 85.3% symmetrical identity with the *Pantoea dispersa* genome. Seventy-five tRNA, 29 rRNA (8 5S rRNA, 8 16S rRNA, 13 23S rRNA), and 7 noncoding RNA (ncRNA) genes and class C and D β-lactamases have putative functions assigned on the basis of the annotation.

### Accession number(s).

This whole-genome shotgun project has been deposited at DDBJ/ENA/GenBank under the accession no. NHZE00000000. The version described in this paper is NHZE01000000.
